# Practical Notes on Diagnosis and Treatment

**Published:** 1906-11-03

**Authors:** 


### Practical Notes on Diagnosis and Treatment.

**Persistent Optic Neuritis.**

"I have been asked to operate on a patient with a lateral tumour of the cerebellum who had been known to have had optic neuritis for nine years, and last year I did operate on such a patient who had been known to have optic neuritis for thirteen years."—*Sir Victor Horsley.*

**Sodium Benzoate in Acute Nephritis.**

Sodium benzoate in doses of 10 to 20 grains is useful in acute Bright's disease. It acts as a mild diaphoretic, a mild diuretic, and as a disinfectant to the urinary tubules. A good combination is tincture of jaborandi ♏. xxx, sodium benzoate, grs. xv., chloroform water to 1 ounce.—*Dr. Gordon Gullan.*

**Arsenic in Leukæmia.**

Dr. Lindsay Dickson reports a case of leukæmia, in a patient of thirty-three years, in which complete recovery followed the free use of arsenic. The initial dose of five minims of Fowler's solution thrice daily was rapidly increased to 15 minims. Under this the enlargement of the spleen and lymphatic glands disappeared and the blood became normal; at the outset the white corpuscles were in numbers almost equal to the red corpuscles.

**Pericardial Effusion in Children.**

It is not very unusual for the distended pericardium to press on the left lung in such a way as to encroach on the vesicular structure and to produce physical signs — dull percussion and tubular breathing—which strongly suggest pneumonic consolidation. If with this there is pyrexia the distinction between the two conditions may be very difficult. Perhaps the absence of any disturbance of the pulse-respiration ratio in the case of pericardial effusion may give a hint against a diagnosis of pneumonia.—*Dr. W. H. Syers.*

**Choroiditis in Children.**

In the choroiditis of young children pigmentation is not a marked feature. When due to inherited syphilis the prolonged use of mercury (two to five grains daily) may both clear up vitreous opacities and bring the choroidal disease to a standstill.—*Mr. Sydney Stephenson.*

**Abdominal Pain in Acute Pneumonia.**

The first evidence of acute pneumonia may be severe pain in the abdomen, and this may lead to the belief that the patient has perforated gastric ulcer or intestinal obstruction. The best guides to the correct diagnosis are the rapidity of the respiration, the initial rigor, and the physical signs in the lungs.—*Dr. Hale White.*

**Headache in Cerebellar Tumours.**

In cerebellar tumours the headache is often occipital, and may be more severe on the side of the tumour when the growth is uni-lateral. But in other cases the pain, instead of being occipital, is referred to the fronto-temporal region. More important than pain in the localisation of a tumour is tenderness, and in the case of a cerebellar tumour help may be derived from local tenderness on pressure in the occipital region.—*Dr. Risien Russell.*

**Gumma of the Brain.**

A good illustration of the value of prompt and large doses of potassium iodide in severe syphilis of the central nervous system is afforded by a case reported by Dr. F. H. Jacob. The symptoms included hemiparesis, loss of sphincter control, semi-unconsciousness, and optic neuritis. The iodide was given in doses of one dram thrice daily with one-sixteenth of a grain of perchloride of mercury; mercurial inunction was also practised. The patient made a good recovery.

